# Recent advances in electrochemical determination of anticancer drug 5-fluorouracil

**DOI:** 10.5599/admet.1711

**Published:** 2023-04-19

**Authors:** Totka Dodevska, Dobrin Hadzhiev, Ivan Shterev

**Affiliations:** Department of Organic Chemistry and Inorganic Chemistry, University of Food Technologies, Plovdiv, Bulgaria

**Keywords:** Electrochemical detection, electroanalysis, pharmaceutical electrochemistry, sensor, analysis, cancer

## Abstract

Reliable, rapid, highly selective and sensitive analytical methods for the determination of antineoplastic agent 5-fluorouracil (5-FU) in human body fluids (blood serum/plasma and urine) are required to improve the chemotherapy regimen to reduce its toxicity and improve efficacy. Nowadays, electrochemical techniques provide a powerful analytical tool for 5-FU detection systems. This comprehensive review covers the advances in the development of electrochemical sensors for the quantitative determination of 5-FU, mainly focused on original studies reported from 2015 to date. We have summarized recent trends in the electrochemical sensor systems applied for the analysis of 5-FU in pharmaceutical formulations and biological samples, and critically evaluated the key performance metrics of these sensors (limit of detection, linear range, stability and recovery). Challenges and future outlooks in this field have also been discussed.

## Introduction

5-fluorouracil (5-FU) is a gold-standard antineoplastic agent used in the chemotherapy of solid tumors (colorectal, breast, head, neck, stomach, pancreatic, and cervical cancer). Antimetabolite drugs work by inhibiting essential biosynthetic processes or being incorporated into macromolecules, such as DNA and RNA, and inhibiting their normal function. The fluoropyrimidine 5-FU does both [1]. After intravenous bolus administration, approximately 80 % of 5-FU is catabolized in the liver into pharmacologically inactive metabolite 5-fluoro-5,6-dihydrouracil (5-FUH2) via dihydropyrimidine dehydrogenase (DPD), 5–20 % of 5-FU are excreted in the urine, and only 1–3 % contribute to the anabolism pathway responsible for clinical effects [2]. The mechanism of action of 5-FU is based on anabolic conversion into cytotoxic nucleotides using several pyrimidine metabolic pathway enzymes [3]. Anabolic way produces 5-fluorouridine-5′-monophosphate (FUMP), 5-fluorouridine (5-FUrd), 5-fluoro-2′-deoxyuridine (5-FdUrd) and their derivatives, responsible for 5-FU cytotoxicity in host and tumor cells [4].

The antitumor activity results from the inhibition of thymidylate synthase – an enzyme required for de novo pyrimidine synthesis. 5-fluoro-2′-deoxyuridine 5′-monophosphate (5-FdUMP) inhibits thymidylate synthase, resulting in the depletion of thymidine triphosphatase (TTP), one of the four nucleotide triphosphates used in the in vivo synthesis of DNA. Other 5-FU metabolites incorporate into both RNA and DNA; incorporation into RNA results in major effects on both RNA processing and functions.

The 5-FU-associated adverse effects include diarrhea, vomiting, nausea, pyrexia, neutropenia, pulmonary embolism, mucosal inflammation, asthenia, and a decrease of haemoglobin level [5]. Leukopenia, leading to an immunocompromised state in these patients, can result in secondary pneumonia or sepsis.

Topical 5-FU has been used in the treatment of several premalignant and malignant skin diseases. It is used for treating multiple actinic or solar keratoses to prevent progression to squamous cell carcinoma in high-risk individuals. 5-FU is usually administered by application directly to the skin lesions as a 0.5 to 5 % cream or solution (common brand names: Carac, Efudex, Fluoroplex, Tolak) twice daily for 14 to 42 days. Due to limited absorption, the systemic side effect profile of topically applied 5-FU is minimal. The most common adverse effects are localized skin irritation, erythema, crusting, and eczematous skin reactions.

There is a large interindividual variability in the clinical outcome (efficacy and toxicity) of chemotherapy based on 5-FU. The effects of 5-FU are strongly dependent on the balance between anabolism and catabolism and, therefore, on the various factors involved in these two phases, such as the substrate concentration, the level of enzymes in the various tissues and their enzymatic activities [6].

Therapeutic drug monitoring (TDM) is the measurement of drug concentrations in biological samples to individualize the drug dosage to improve drug efficacy and reduce related toxicities. TDM is especially important for patients with DPD deficiency, in which 5-FU treatment is the mainstay of treatment for their condition with no clinically efficacious alternatives. Patients may have a partial or complete deficiency of DPD and be more likely to experience toxicity with 5-FU. Conversely, some patients may also have increased DPD activity, which may result in reduced exposure to 5-FU [7].

Over the past years, increasing efforts have been placed on optimizing 5-FU dosing to increase antitumor efficacy while minimizing drug-associated toxicity. There is growing evidence to show that 5-FU dosing based on plasma 5-FU drug level is feasible and that 5-FU TDM can improve clinical outcomes by improving the efficacy of 5-FU-based combination regimens and reducing toxicities [8]. Casale *et al.* 2004 confirmed the relevance of the pharmacokinetic analysis of 5-FU main metabolites, especially 5-FUH2, to better understand the metabolism and improve the therapeutic efficacy [9]. The results from the commented study provided evidence that the 5-FUH2/5-FU ratio calculation should be used as reference values to decide and monitor optimal dose scheduling for the decrease of toxicity and the improvement of therapeutic efficacy.

Reliable analytical techniques for monitoring 5-FU concentrations in biological fluids after intravenous administration effectively improve the chemical agent's therapeutic index. Here it should be noted that blood samples containing 5-FU require more complex handling than samples for most other drug analyses since 5-FU continues to degrade in blood once the sample is taken. Two approaches are used to stop enzymatic degradation: placing the sample immediately in ice and storing it on ice until further processed in the laboratory or adding a small amount of a DPD enzyme inhibitor immediately after collection [7]. This was observed to remove the need for immediate centrifugation and separation of plasma.

In physiological samples of clinical patients, the mean concentration of 5-FU varies over a wide concentration range of 0.106 to 3.77 μg mL^–1^ (0.82 to 29.2 μM) in serum samples and as high as 10 to 60 μg mL^–1^ (77.4 to 464.2 μM) in urine samples [10]. The analytical methods developed for the determination of 5-FU in biological matrices have been reviewed extensively by Breda & Barattè [3]. In 2020 Semail *et al.* presented an updated review on the analytical method development and validation of 5-FU and its metabolites in biological matrices [11]. The article primarily addressed the development of sample preparation methods, analytical methodologies and the summary of sensitivity and recovery of the compounds from 1980 to February 2020. Authors presented a review on chromatographic and electrophoretic systems, which are the most commonly used strategies for the analytical separation of 5-FU and its metabolites from samples.

Monitoring of 5-FU can be performed using high-performance liquid chromatography (HPLC), gas chromatography-mass spectrometry (GC–MS) or liquid chromatography-mass spectrometry (LC–MS). HPLC–UV detection is the most frequently reported technique for the determination of 5-FU in plasma, serum, whole blood, tissue and urine. Comparing the operational parameters of different techniques for the determination of 5-FU in plasma, Breda & Barattè conclude that LC–MS is the most sensitive technique; GC-MS and GC are almost similar in sensitivity to LC-MS, but these approaches require a time-consuming, tedious extraction and derivatization steps [3]. Generally, the described techniques needed a relatively large plasma volume (2.0 mL), which may not always be available, required sophisticated equipment and are not amenable to rapid and routine clinical assay.

Therefore, simple, rapid, cost-effective, easily miniaturized, highly selective, sensitive and properly validated analytical methods for the determination of 5-FU in human body fluids (blood serum/plasma and urine) are required to improve the chemotherapy. Further, controlling the amount of 5-FU in commercial formulas is vital in the pharmaceutical industry quality control.

The received data testify that the electrochemical techniques can offer the advantages mentioned above and provide a feasible path toward the next generation of sensing devices. To the best of our knowledge, there is no review article related to the electrochemical determination of 5-FU. The next parts of the manuscript present the most recent and innovative works on the fabrication of electrochemical sensor platforms and their application for 5-FU determination in biological and pharmaceutical samples. We have comprehensively reviewed the most promising sensor designs and successfully applied them for the electroanalysis of 5-FU, mainly focused on original studies reported from 2015 to date.

## Basic detection principles of the most common electrochemical sensing techniques

Various electrochemical techniques – chronoamperometry, cyclic voltammetry (CV), linear sweep voltammetry (LSV), staircase voltammetry (SCV), differential pulse voltammetry (DPV), square wave voltammetry (SWV), and square wave adsorptive stripping voltammetry (SWAdSV) can be used as an efficient alternative, providing an affordable approach for an accurate, highly selective, sensitive, and fast quantitative determination of 5-FU. The basic detection principles of the most common electrochemical sensing techniques are summarized in Table 1.

## Electrochemical sensors for 5-FU determination

**Figure 1.**
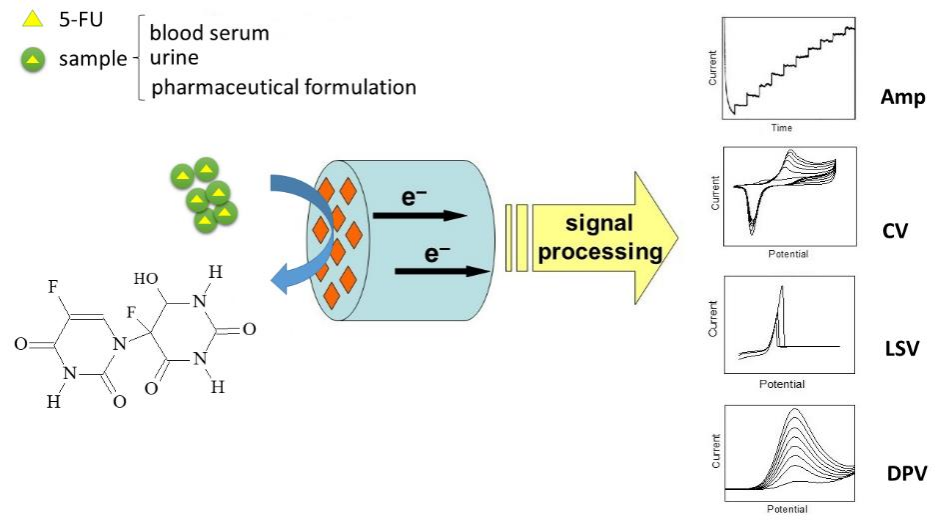
Illustrative representation of electrochemical detection of 5-FU.

The general principle of electrochemical detection of 5-FU is illustrated in Fig. 1.

Earlier electrochemical methods used for the quantitative determination of 5-FU include the use of mercury electrode. Mirčeski *et al*. 2000 studied the redox reaction of 5-FU at a hanging mercury drop electrode (HMDE) by means of SWV [12]. The effect of the Cu(II) ions on the adsorptive SWV response of 5-FU was also discussed from an analytical point of view. Authors demonstrated that in the presence of Cu(II) ions, SWV provides the determination of 5-FU below nano-molar concentration levels. 5-FU reacts with Cu(II) yielding a stable complex. The complex formed is adsorbed more effectively at the electrode surface, increasing the adsorptive stripping response of 5-FU. A linear calibration plot was established at 1-9×10^−11^ M with a correlation coefficient of *R*^2^ = 0.992 and an extremely low detection limit of 7.7×10^−12^ M was achieved.

The development of new, cost-effective, efficient electrocatalysts plays a key role in the design of high-performance electrochemical sensing devices. The great potential of carbon-based electrodes as sensing platforms is exciting due to their unique electrical and chemical properties, such as a high surface-to-volume ratio, high electrical conductivity, chemical stability, biocompatibility, and robust mechanical strength [13,14].

Carbon nanomaterials (carbon nanotubes, graphene, carbon quantum dots, carbon nanofibres, *etc.*) have exhibited such inherent features that can be easily exploited in the development of novel advanced technology for sensing applications [15,16]. Carbon nanotubes (CNTs) offer excellent properties that enable a wide range of applications in electrocatalysis. CNTs may be comprised of a single graphitic layer or multiple coaxial layers, resulting in the formation of single-walled carbon nanotubes (SWCNTs) and multi-walled carbon nanotubes (MWCNTs). The variable surface morphology of CNTs permits a great variety of surface functionalities for the development of reliable and stable electrochemical sensor platforms. The electrochemical sensing performance (sensitivity and selectivity) of the CNTs can be modified by the covalent or non-covalent functionalization of CNTs [15].

Graphene is a two-dimensional (2-D) sheet of hexagonally arranged carbon atoms with sp^2^-hybridization. The combination of attributes such as an extremely large surface area (at 2630 m^2^ g^-1^, it is double that of SWCNTs), very large 2-D electrical conductivity, high mechanical strength, and low cost makes graphene an ideal platform for the anchoring of metal nanoparticles for electrochemical sensing applications [14,17]. There are excellent examples of applications of graphene decorated with catalyst NPs for electrochemical sensing [17,18].

Graphene oxide (GO) contains oxygen functional groups, including hydroxyl, carboxylic, and epoxy groups, which demonstrate good catalytic activities, particularly for the oxidation of organic molecules. However, the presence of oxygen-containing groups disrupts the *s*p^2^-bonds of carbon rings, thereby lowering conductivity. This issue may be resolved by reducing graphene (electrochemically, thermally, or chemically) to generate reduced graphene oxide (rGO). This form of graphene exhibits higher conductivity due to the cleavage of hydroxyl and epoxy functional groups while retaining most of the carbonyl or carboxylic acid groups at its edges [19].

Since the carbon nanomaterials-based sensors are unable to differentiate between the responses of organic compounds with the same functional group in their structures, the accurate quantification of the target analyte becomes difficult. In order to enhance the electrochemical sensing performance of carbon nanomaterials, a mixture or hybrid or composite of these nanomaterials with organic or inorganic compounds has to be used [15]. The addition of some other materials, such as nanoparticles of metals or metal oxides, functionalized nanostructures, as well as organometallic compounds, is an appropriate approach to further enhance the sensitivity and lower the limits of detection for the target analyte through synergistic effects.

A high-quality review article [20] surveys state-of-the-art nanomaterials-based electrochemical sensors and biosensors for the detection and quantification of six classes of important pharmaceutical compounds, including anti-inflammatory, anti-depressant, anti-bacterial, anti-viral, anti-fungal, and anti-cancer drugs. A critical review focused on the recent advances in metal nanocomposite-based electrochemical sensors for pharmaceutical analysis was published in 2022 [21]. The authors have presented a comprehensive overview of representative metal nanocomposites with synergistic properties and their recent (2017–2022) application in the context of electrochemical sensing as a means of detecting specific antibiotic, tuberculostatic, analgesic, antineoplastic, anti-psychotic, and anti-hypertensive drugs.

Hua *et al*., 2013 reported the electrochemical behavior of 5-FU on a glassy carbon electrode (GCE) modified with bromothymol blue (BTB) and MWCNTs [22]. Quantitative analysis of 5-FU was performed in 0.2 M PBS (pH 6.8) using cyclic voltammetry (CV) and a linear relationship between the oxidation peak current and logarithmic concentration of 5-FU in the concentration range of 8×10^−7^ to 5×10^−3^ M was obtained (LOD = 2.67×10^−7^ M). The results of the RSD (2.8 %) and recovery (98.6 %) showed that this proposed method can be used efficiently for the determination of 5-FU in the pharmaceutical formulation. The authors point out that the electrode surface was renewed electrochemically after each experiment to minimize electrode fouling and improve the electrochemical response.

Two years later, a fast and facile electrochemical sensor for the detection of *ppb* levels of 5-FU with an analysis time as low as 25 s was developed by Satyanarayana *et al.* [10]. The sensing platform was fabricated using a gold nanoparticles-decorated multiwall carbon nanotubes composite modified GCE. The electrochemical capability of the fabricated AuNPs-MWCNTs-Chit/GCE for the detection of 5-FU is examined by CV, electrochemical impedance analysis and DPV. Peak current of the DPVs exhibited a linear relationship over a wide concentration range of 0.03–10 μM with a low detection limit of 20 nM (2.6 ng mL^–1^). The recovery values obtained in these experiments varied from 98.3 to 102.6 % and are quite satisfactory. The authors have also provided data about the storage stability and reusability of the developed electrode. DPVs of 20 μM 5-FU were recorded at a single AuNPs-MWCNTs-Chit/GCE electrode over a period of 7 days while storing the modified electrode at ambient laboratory conditions. The peak currents of the recorded DPVs decreased merely by 3.8 % in about 25 measurements. Practical utility of the fabricated sensor has been demonstrated for the detection of 5-FU directly from artificial urine and pharmaceutical formulations with good recovery limits and clearly confirms that the developed sensor would be of biomedical interest.

A glassy carbon electrode modified with a chemically reduced graphene oxide and chitosan (CRGO/CS) composite film was constructed and applied to determine 5-FU using SCV and SWV techniques [23]. The sensor CRGO/CS/GCE showed a lower detection limit (1.24 nM for SCV, and 4.93 nM for SWV) and it was successfully applied to determine 5-FU in real samples. The proposed method was validated for the quantitative determination of 5-FU in pharmaceutical preparations. It was used to detect 5-FU in Florac Injection (50 mg ml^–1^) by applying SCV using the standard addition method. Additionaly, the applicability of the CRGO/CS/GCE was checked by analyzing 5-FU in spiked urine samples. In these studies, authors report high recovery values (97.3 to 100.7 %) and low RSD values (0.79–1.15 %), revealing the accuracy and precision of the proposed method.

The electrochemical behavior of 5-FU was investigated at a self-made MWCNTs-paraffin oil paste electrode using CV and DPV [24]. The oxidation was irreversible over the pH range studied (pH = 3.0 to 11.2) and exhibited a diffusion-controlled process. In the range of 10^–7^ to 5×10^–6^ M, the current measured by DPV presents a good linear property as a function of the concentration of 5-FU with a detection limit of 3.94×10^–8^ M. The proposed method was validated for the determination of 5-FU in pharmaceutical preparations in “Aduracil” tablets as a real sample by applying DPV using the standard addition method and the results were in good agreement with the content marked in the label. The recovery test of 5-FU ranging from 4.0 μM to 1.0 μM was performed using DPV. The recoveries in different samples were found to lie in the range from 97.15 to 101.82 %, with RSD = 2.09 %. Here it should also be noted that during the successive cyclic voltammetric sweeps, a significant decrease in the oxidation peak current was noticed. This phenomenon may be attributed to the fouling of the electrode surface due to the adsorption of the oxidation products and severely affects the analytical performance in terms of sensitivity and linearity of the electrode signal. Authors noted that the current response of the prepared electrode would decrease after successive use and the electrode should be prepared again. The established feature would limit the use of this sensor for 5-FU analysis. Therefore, future efforts should be focused on appropriate reverse electrode passivation materials to reduce the effect of fouling.

Carbon paste electrodes (CPEs) are widely applicable in electrochemical studies. CPEs have multiple beneficial features and practical advantages including excellent electric properties, low cost, easy preparation, simple regeneration of their active surface and possibilities of miniaturization. Feasibility of incorporating different substances during the paste preparation makes CPE a strong tool to evaluate different modifiers, building a useful platform for many electrochemical applications.

Methylene blue (MB) is a cationic dye whose electrochemical properties are well known. MB-modified CPE as a sensor for the voltammetric investigation of 5-FU was reported by Bukkitgar & Shetti [25]. Due to the excellent property of MB as a redox mediator, improved and enhanced results were obtained compared to the unmodified electrode. The peak currents registered with DPV, were linear with the concentration of 5-FU in the range of 10^−7^ to 4×10^−5^ M and the detection limit was calculated to be 2.04 nM. Simple procedure for preparation and regeneration of surface with good reproducibility, high sensitivity, and low detection limit implied the applicability of the proposed electrode for 5-FU determination. Under the optimum experimental conditions, the authors evaluated the effects of potential interferents on the voltammetric response of 0.1 mM 5-FU. The experimental results showed that 100-folds of citric acid, gum acacia, oxalic acid, sucrose, xanthine, uric acid and urea did not interfere with the voltammetric signal of 5-FU. However, when caffeine and ascorbic acid were used, peak potential showed remarkable variation, indicating that these substances will have an interfering effect on the quantitative detection of 5-FU.

Nanostructures of lanthanides (or *f*-block metallic elements) have interesting electrocatalytic properties and can be used as modifiers in sensors. CPE modified with praseodymium erbium tungstate nanoparticles (Pr:Er NPs) was also investigated for the voltammetric detection of 5-FU [26]. An irreversible 5-FU oxidation peak was registered at 1.0 V (*vs.* Ag/AgCl) in 0.01 M PBS (pH 7.0). A linear calibration was achieved in the range of 0.01 to 50 μM (LOD = 0.98 nM) using SWV. The authors stated that the sensor provided good precision and accuracy. The practical applicability of developed electrode material Pr:Er/CPE to detect selectively 5-FU in spiked urine and plasma samples was tested. The results obtained from the developed sensor satisfactorily agreed with HPLC, indicating the reliability of the electrochemical method.

Researchers have used various types of ionic liquids (ILs) in electrode fabrication with the intention of developing novel electrochemical sensor platforms or as unique electrocatalytic substances, as they possess many attractive properties: sophisticated conductivity, low volatility, high chemical stability, and wide electrochemical windows [27]. Therefore, ILs can be used as modifiers to make a new kind of modified carbon paste electrodes (IL-CPE), which have exhibited advantages including improved electrocatalytic ability, anti-fouling effect and good stability.

For the first time, the electrochemical oxidation of 5-FU on an IL-CPE was investigated by Zhan *et al.* [28] IL-CPE was fabricated using 1-butylpyridinium hexafluorophosphate (BPPF6) as the modifier. Quantitative analysis of 5-FU was performed in B-R buffer solution (pH 7.0) using differential pulse voltammetry and two linear ranges (5×10^−7^ to 2×10^−6^ M and 2×10^−6^ to 8×10^−4^ M) were obtained; LOD was found to be 1.3×10^−8^ M. The electrode was stored at room temperature when not in use, it could remain 95.9 % of its initial responses after storage for 15 days, indicating that IL-CPE had good storage stability. Furthermore, to demonstrate the feasibility of the IL-CPE, the amounts of 5-FU in commercial injection samples were tested by DPV, which showed good recovery (in the range from 97.2 to 102.1 %) and practical applicability.

Combined nanomaterials and ILs have synergistic effects and result in improved conductivity, active sites, and accelerated electron transfer rate. In these cases, multiple-amplifying voltammetric response could be determined and increased sensitive analyte detection would be possible. A novel sensitive electrochemical approach was developed by incorporating graphene quantum dots (GQD) and ionic liquid 1-butylpyridinium bromide (BPBr) in the fabrication of a carbon paste electrode (GQD/BPBr/CPE) [29]. The applicability of the GQD/BPBr/CPE in the voltammetric analysis of 5-FU was evaluated. In square wave voltammetry analysis, the GQD/BPBr/CPE showed high selectivity and sensitivity for 5-FU over a wide linear range of 0.001 to 400 μM. Owing to the synergic effect of GQD and BPBr, an extremely low detection limit of 0.5 nM (*S*/*N*=3) was achieved. The oxidation current of 5-FU at GQD/BPBr/CPE showed 92.3 % of its original signal after 14 days, confirming the good stability of the modified electrode. The GQD/BPBr/CPE was successfully applied for the determination of 5-FU in pharmaceutical samples and acceptable results were obtained.

The composition of carbon nanotubes, copper(II) oxide nanoparticles and ionic liquid loaded on CPE, which acted as part of highly sensitive and selective electrode for the analysis of two anticancer drugs, was described by Fouladgar [30,31]. The electrode was fabricated using 1-ethyl-3-methylimidazolium tetrafluoroborate (EMITFB). Doxorubicin and 5-FU can be oxidized at potentials of approximately 0.68 and 0.97 V (*vs.* Ag/AgCl) on EMITFB/CuO-SWCNTs/CPE surface that is sufficient for the simultaneous determination of drugs in a mixture via SWV [30]. The relationship between current and concentration was linear within the range 160 to 760 μM (LOD = 6.0 nM) for doxorubicin and 20–180 μM (LOD = 0.4 μM) for 5-FU. The sensitivities of the modified electrode toward doxorubicin in the absence and presence of 5-FU were virtually similar, thus indicating that the oxidation of doxorubicin and 5-FU at the modified electrode are independent and simultaneous or independent measurement of the two compounds are possible. The application of EMITFB/CuO-SWCNTs/CPE for the quantitative analysis of doxorubicin and 5-FU in real samples (serum and commercial pharmaceutical samples), produced favourable outcomes.

One year later, Roushani *et al.* reported CuNPs/MWCNTs/IL/Chit/GCE, fabricated using 1-methyl-3-octylimidazolium tetrafluoroborate ionic liquid [32]. The sensor has similar characteristics – the peak current of the DPV exhibited a linear relationship against 5-FU over a concentration range of 1–110 μM with a detection limit of 0.15 μM. The modified electrode demonstrated satisfactory long-term stability – it retains 95 % of the initial response after two weeks storage and 93 % after 30 days, respectively. Using the standard addition method the concentration of 5-FU in an artificially prepared specimen, by adding known amounts of 5-FU to serum samples, was measured. The obtained recovery (89.05–104.9 %) reveals the potential capability of the method for the determination of 5-FU in human blood serum samples.

Recently, green nanotechnology has remained at the forefront of scientific research due to its outstanding approaches and applications. Green nanotechnology involves the application of green chemistry principles to the design of valuable and sustainable nano-sized materials in a more environmentally benign approach. The so-called “green synthesis”, using mild reaction conditions and natural resources as plant extracts, has received more attention as a cost-effective and valuable alternative for environmentally safe and energy-efficient production of metal nanoparticles [33]. There is convincing evidence that bio-inspired synthesis of metal nanoparticles has the potential to provide a new direction in the fabrication of novel, cheap and effective electrocatalytic materials applicable to electroanalysis.

Lima *et al*., 2018 reported for the first time utilization of porphyran (PFR) (a sulfated polysaccharide extracted from red seaweed) as a capping, stabilizing, and reducing agent for the cost-effective and environmentally safe synthesis of AgNPs and their applicability for the development of an electrochemical sensor for 5-FU determination [34]. The fabricated electrochemical platform based on AuNPs-PFR/CPE, combined with the DPV technique, exhibits a linear current response in the concentration range from 29.9 to 234.4 μM, LOD of 0.66 μM, and LOQ of 2.22 μM, respectively. The good analytical performance of the sensor was confirmed for determining 5-FU in pharmaceutical formulation (commercial injection solution), with good recoveries (96.6 to 101.4%) and an acceptable relative standard deviation (RSD=2.80 %). Unfortunately, the authors do not provide data on the long-term storage stability of the modified electrode.

In conclusion, although these synthetic methods restrict the use of toxic chemicals, energy and sophisticated instruments, the experimental data show that the electrode surfaces modified with bio-synthesized metal nanoparticles still remain challenging as they are not as stable and reproducible as one would hope. Further research needs to be done to improve the electrode performance in terms of operational and storage stability of the electrode-catalysts modified with nanoparticles synthesized in a green way.

A novel class of chitosan-based hybrid semi-interpenetrating nanogel catalyst by the reductant-free synthesis of AuNPs within the chitosan-poly(methacrylic acid) (Au@CS-PMAA) polymer network was reported [35]. The covalently cross-linked Au@CS-PMAA nanogel with CS chains semi-IPN in the PMAA cross-linked network shows superior colloidal stability, increased catalytic activity and proved to be an effective electrochemical sensing platform for 5-FU. Under optimal conditions, the constructed sensor Au@CS-PMAA/GCE offered good analytical performance in the determination of 5-FU with a wide linear range from 0.1–497 μM and a detection limit of 0.03 μM. In addition, the peak current response of the Au@CS-PMAA/GCE retains 92.5 % of the initial peak current value after storage of 30 days, revealing good long-term stability of the modified electrode. DPV measurements in diluted human blood serum samples prove the practical applicability of the sensor.

Here it should be pointed out that some drawbacks, such as low homogeneity and reproducibility of the resulting modified surface, could characterize the drop-casting approach used in [10,22,35]. The influence of the “coffee ring” effect can alter the distribution of the nanoparticles drop-casted on an electrode. However, voltammetry requires the formation of uniformly modified surfaces for which the coffee ring and its related effects present a significant limitation on the reproducibility of the drop-casted surfaces [36]. Moreover, the modifier that has been physically adsorbed onto the electrode surface may be gradually stripped off in long-term measurements.

Thermo-sensitive conductive polymer microgels are unique materials applicable in the fields of electronics, medical electrodes, soft robotics, and electrochemical energy storage devices. Among all stimulating responsive polymer microgels, thermo-sensitive poly(N-isopropylacrylamide) (PNIPAM) attracts an extensive range of scientists interested in the field of sensors due to its lower critical solution temperature (around 32 °C), close to the human body temperature. Various multifunctional PNIPAM systems have revolutionized biomedical fields, such as controlled drug delivery, tissue engineering, and self-healing materials [37]. In the field of electroanalysis, thermo-sensitive conductive polymer forming a charged smart membrane by cycling “on-off” over the surface of the electrode allows for a “command interface” that selectively enhances or inhibits the electrochemical reaction [38].

A new type of switch-like temperature-controlled 5-FU electrochemical sensor based on the adaptable thermosensitive conductive microgel consisting of PNIPAM and poly(3,4-ethylenedioxythiophene) (PEDOT) was developed [39]. Chen *et al.* constructed a smart electrochemical 5-FU sensor with temperature control using the adaptable conductive polymer microgel PNIPAM-PEDOT containing electrically conductive and thermoresponsive properties. The resulting conductive microgel exhibits a thermo-reversible conversion by controlling the internal temperature in the range of 20–40 °C, owing to the contracted and expanded configuration of the PNIPAM. The sensor was successfully used to achieve thermo-switch-control for detection of 5-FU for the first time. An effectively switched the electrochemical process by tuning the solution temperature and achieved sensitive quantitative determination of 5-FU. The PNIPAM-PEDOT/GCE has good selectivity and reproducibility for 5-FU detection. Furthermore, the sensor demonstrated satisfactory results toward 5-FU detection in real human blood serum samples. Authors concluded that the presented novel switch-like electrochemical sensor offers an innovative notion for the application of thermo-responsive polymers.

Ratiometric electrochemical sensors (RECSs) have attracted extensive attention because of RECSs significantly reduce the interference of the inherent background electrical signals, as well as possess great advantages in terms of enhanced accuracy, repeatability and reliability. RECSs are capable of overcoming the system errors of the conventional electrochemical sensors derived from the alteration of the environment and operating personnel [40]. The RECS possesses dual electrochemical signals and the quantitative detection of the target analyte is based on the ratio of these two signals. By applying the ratiometric method, a built-in correction coefficient is created to reduce the influence of general interfering species.

Hatamluyi *et al*., 2021 reported a ratiometric electrochemical sensor based on GCE modified with Pd-Au/MWCNTs-rGO nanocomposite for simultaneous quantification of Irinotecan (IRI) and 5-FU in both real samples and aqueous solutions [41]. Introduction of Pd-Au/MWCNT-rGO significantly improved the speed of electron transport, specific surface area, and electrical catalytic ability of the sensing system due to the synergistic effect of Pd-Au bimetallic nanoparticles and MWCNT-rGO hybrid structure. The assay strategy was based on the use of a fixed concentration of ferrocene (Fc) added to the electrolyte solution (Robinson buffer solution). Fc is a built-in effective internal reference to achieve ratiometric detection of IRI and 5-FU. DPV, registered after the addition of IRI and 5-FU, clearly shows three separated and independent characteristic peaks appeared at +0.2, +0.58, and +1.17 V (*vs.* Ag/AgCl, sat. KCl), which were ascribed to the oxidation of Fc, IRI, and 5-FU, respectively. Under optimal conditions, a linear relationship between the IRI and 5-FU concentrations and ratiometric peak current intensities (*I*_IRI_/*I*_Fc_) and (*I*_5-FU_/*I*_Fc_) was obtained. By measuring the (*I*_IRI_/*I*_Fc_) and (*I*_5-FU_/*I*_Fc_) as the response signals, simultaneous detection of IRI and 5-FU were realized with excellent selectivity and reproducibility in contrast with the single-signal method. Based on the developed ratiometric method, sensitive and reliable detection of 5-FU has been realized in the dynamic range 0.05 to 75 μM (LOD = 9.4 nM). The authors have provided data on the long-term stability of Pd–Au/MWCNT-rGO/GCE. Six electrodes from the same batch were stored at 4 °C for 6 weeks and applied every week. After 42 days, the original value of *I*_5-FU_/*I*_Fc_ exhibited negligible changes – the RSD was estimated <2.1 %, representing acceptable stability. In the analysis of real biological samples (injection solution forms of IRI and 5-FU, human urine and serum samples), the sensor possesses high detection recoveries and low RSDs.

The advanced electrochemical sensor platforms for the detection of 5-FU and their applications in the clinical and pharmaceutical fields are listed in Table 2. Considering the serious impacts of 5-FU on human health, the here presented novel electrochemical sensors is expected to hold great potential for fast, simple, and on-site 5-FU detection in blood serum and pharmaceutical formulations.

## Electrochemical sensors – advantages and limitations

Electrochemical sensor platforms are ideally suited for rapid and reliable point-of-care sensing of 5-FU. The methods for 5-FU detection have advantages over the spectrophotometric and chromatographic methods mostly because of the ease of conducting experimental work and the cost-effectiveness of the instrumentation.

Chromatography-based techniques (GC, HPLC) and hyphenated approaches (HPLC-UV, HPLC-MS, GC-MS), the most recent techniques used for detection of 5-FU, have require relatively great investment and expertise, as well as complicated working operation including multistage sample preparation – tedious extraction and derivatization procedures. The high hydrophilicity of 5-FU and its active metabolites hinders their isolation from biological matrices. The high aqueous solubility and the low solubility to organic solvents result in extraction difficulties [48,49]. The most applied techniques to remove interfering endogenous substrates, namely liquid-liquid extraction (LLE), solid-phase extraction (SPE) and protein precipitation (PP), have been thoroughly reviewed [49]. The main drawback of such techniques is high consumption of organic solvents. Extraction usually employs the acidification of serum sample as well as PP (using trichloroacetic acid, mixtures of methanol and water, or mixtures containing acetonitrile) followed by LLE (using different solvents such as propanol, methanol, ethyl acetate, diethyl ether, or chloromethane). Currently, more attention is being paid to the development of efficient and environmentally friendly microextraction methods that consume a much lower amount of organic solvents.

Moreover, MS detection is incompatible with microdialysis samples in terms of signal stability because the dialysis salts can induce high background noise. The upkeep of accessory instruments is expensive and the high cost makes the MS detector, coupled with HPLC and GC, less widely used.

Here it must be emphasized that the differences in sample pre-treatment, experimental conditions and instrumental related parameters can cause variations in 5-FU concentrations detected.

In contrast, the electroanalytical techniques are quick, simple and offer the possibility of direct analysis. Electrochemical sensing is the only method that allows the determination in an aqueous medium without derivatization of the analyte. It should be considered that electrochemical sensors have an enormous linear dynamic range of more than six orders of magnitude and exhibit a high degree of selectivity. The possibility of regenerating the catalytic active electrode surface allows numerous analyses in succession. Unlike their spectroscopic and chromatographic counterparts, electrochemical sensors may be incorporated into robust, portable or miniaturized devices while remaining relatively inexpensive and simple compared to spectrophotometric detectors or mass spectrometry.

Table 3 highlights the advantages and disadvantages of electrochemical sensor devices. Although there are still some drawbacks to using this type of equipment, advantages exceed shortcomings.

Electrochemical sensors for 5-FU demonstrate irreplaceable advantages in biomedical and clinical applications, including easy-to-operate, rapid response, excellent detection limits and ability to handle small sample volumes. Thus, the electrochemical sensors for 5-FU have a high potential to outcompete traditional methods in the near future.

## Conclusion remarks and outlook

The outstanding features of electrochemical sensor devices have brought many prospects in clinical and pharmaceutical applications. High sensitivity, selectivity, portability and affordable investment provide reliable alternatives to the conventional analytical methods used in clinical labs. Moreover, electrochemical sensors hold great promise for enabling clinical analysis of 5-FU at the point-of-care.

Transforming an electrochemical sensor system into a simple, easy-to-use, and portable device from lab-scale research is the driving force for the commercialisation of sensor systems. Although most of the electrochemical sensors discussed here were tested in real samples analysis, there is a considerable gap between the laboratory tests and the fabrication of commercial sensing devices for practical applications. There are still shortcomings and further research is required for their overall improvement. Some issues, such as unsatisfactory long-term stability, electrode fouling and the non-specific adsorption of other species, make the proposed sensors more difficult to apply commercially. Therefore, novel strategies for electrode signal amplification are needed to be developed. Here we summarize the most promising approaches for improving the electrochemical determination of 5-FU**:**

- To achieve reliable measurements, the effects of fouling can be sufficiently minimized by incorporating the electrochemical activation of the electrode surface at regular intervals as a part of the analysis procedure.
- Measuring in flowing systems. The use of flow injection analysis (or batch injection analysis) systems combined with modified screen-printed electrodes will substantially reduce the contact between the electrode and fouling agents. Additionally, in all these systems, products/intermediates of the electrochemical reaction are removed (washed away) from the working electrode, thus minimizing their deposition on the electrode surface.
- Application of protecting films. The working electrodes can be modified with protein, polymeric or nonpolymeric films in order to provide a physical barrier between the fouling agents and the electrode surface.
- Development of novel electrode materials more resistant toward passivation.
- The use of boron-doped diamond (BDD) electrodes is still unexplored in this area of research. BDD is a conductive material effective for real-time detection because the sp^3^-hybridized structure of BDD is resistant to biofouling and biocompatible with organisms. In comparison with other electrode materials (GC, graphite, Pt and Au), BDD possesses a higher resistance toward biofouling, the largest electrochemical potential window for aqueous media, as well as a low and stable background current, which is attributed to the low capacitance of the BDD. All these properties make BDD suitable for in vivo real-time determination of 5-FU.

Additionally, it is essential to develop and validate electrochemical sensors for the simultaneous quantification of 5-FU and its metabolites in human plasma. For clinical practice, the method could successfully improve the effectiveness and security of the chemotherapy regimen.

We are convinced that the joint work of interdisciplinary research groups will contribute to more recent commercialization of high-performance electroanalytical devices for 5-FU determination.

## Figures and Tables

**Table 1.** Basic detection principles of the most common electrochemical sensing techniques.

Electrochemical technique	Detection principle
Amperometry	Amperometry is based on the application of a constant potential to the working electrode, and the subsequent measurement of the current generated by the oxidation/reduction of the electroactive analyte. The resulting steady-state current is proportional to the bulk concentration of the analyte.
CV	The technique is based on varying the applied potential of the working electrode in both the forward (anodic sweep) and the reverse (cathodic sweep) directions while measuring the corresponding current. CV is often used to investigate the reversibility, reaction kinetics, electron stoichiometry of a system, the diffusion coefficient of an analyte, and the formal redox potential.
LSV	The current at a working electrode is measured while the potential between the working electrode and a reference electrode is swept linearly in time.
SCV	The potential sweep is a series of stair steps; the current is measured at the end of each potential change, right before the next. Thus the contribution to the current signal from the capacitive charging current is reduced.
DPV	Short pulses (in the range of 10 – 100 ms) with limited amplitude (up to 100 mV) are superimposed on a linear ramp. The current value is immediately measured before the pulse application and at the end of the pulse. The difference between the currents is recorded and plotted *versus* the potential. The procedure effectively reduces the capacitive current due to the direct current ramp.
SWV	A symmetrical square-wave pulse is superimposed on a staircase wave. The duration of the pulse is equal to the length of the staircase, and the superposition is obtained in such a way that the forward pulse of the square wave coincides with the first half of that staircase. The first current is measured at the end of the forward square-wave pulse, and the second one is measured at the end of the return square-wave pulse. The signal is obtained as an intensity of the resulting differential current. The change in current between potential steps is plotted *versus* the potential.

**Table 2.** Comparison of linear range and LOD values for 5-FU determination using electrochemical sensor platforms.

Electrode	Method	Linear range	Limit of detection	Real sample	Ref.
AuNPs-MWCNTs-CS/GCE	DPV	0.03-10^−6^ M	2210^−8^ M	artificial urine, pharmaceutical formulations	[10]
HMDE	SWV	1-9×10^−11^ M	7.7710^−12^ M	–	[12]
MWCNTs/BTB/GCE	CV	8810^−7^ - 5510^−3^ M	2.67210^−7^ M	pharmaceutical formulation	[22]
CRGO/CS/GCE	SCV	0.01-0.15 μM	1.24 nM	urine	[23]
MB/CPE	DPV	1×10^−7^ - 4×10^−5^ M	2.04 nM	urine	[25]
Pr:Er/CPE	SWV	0.01-50 μM	0.98 nM	blood serum, urine	[26]
IL-CPE	DPV	5510^−7^ - 8810^−4^ M	1.3110^−8^ M	pharmaceutical formulation	[28]
GQD/BPBr/CPE	SWV	0.001-400 μM	0.5 nM	pharmaceutical formulation	[29]
EMITFB/CuO-SWCNTs/CPE	SWV	20-180 μM	0.4 μM	pharmaceutical formulation, blood serum	[30]
CuNPs/MWCNTs/IL/CS/GCE	DPV	1-110 μM	0.15 μM	blood serum	[32]
AuNps-PFR/CPE	DPV	29.9-234.4 μM	0.66 μM	pharmaceutical formulation	[34]
Au@CS-PMAA/GCE	DPV	0.1-497 μM	0.03 μM	blood serum	[35]
PNIPAM-PEDOT/GCE	DPV	0.03-182 μM	15 nM	blood serum	[39]
Pd–Au/MWCNT-rGO/GCE	DPV	0.05-75 μM	9.4 nM	pharmaceutical formulation, urine, blood serum	[41]
AuNP-SPE	SWV	0.2-50 μg mL^−1^	0.1 μg mL^−1^	pharmaceutical formulation	[42]
P(BCP)/dsDNA/GCE	DPV	1-50 mg L^−1^	0.31 mg L^−1^	pharmaceutical formulation	[43]
ZnFe_2_O_4_/MNPs/IL/CPE	SWV	0.1-1400 μM	0.07 μM	pharmaceutical formulation, urine	[44]
AgNPs@PANINTs/PGE	DPV	1.0–300 μM	0.06 μM	blood serum	[45]
GQDs-PANI/ZnO-NCs/GCE	DPV	0.1-50 μM	0.023 μM	pharmaceutical formulation, blood serum, urine	[46]
CuFe_2_O_4_ NPs/SPGE	DPV	0.1-270 μM	0.03 μM	pharmaceutical formulation, urine	[47]

BTB – bromothymol blue; HMDE – hanging mercury drop electrode; SPE – screen-printed electrode; GQD – graphene quantum dots; BPBr – 1-butylpyridinium bromide; PNIPAM – poly(N-isopropylacrylamide); PEDOT – poly(3,4-ethylenedioxythiophene); PMAA – poly(methacrylic acid); CRGO – chemically reduced graphene oxide; CS – chitosan; PFR – porphyran; MB – methylene blue; P(BCP) – poly(bromocresol purple); dsDNA – fish sperm double strand DNA; MNPs – magnetic nanoparticles; PANINTs – polyaniline nanotubes; PGE – pencil graphite electrode; NCs – nanocomposites; EMITFB – 1-ethyl-3-methylimidazolium tetrafluoroborate.

**Table 3.** Summary of advantages and disadvantages of electrochemical sensors.

Advantages	
Selectivity and sensitivity	Electroanalytical techniques are powerful, highly sensitive and selective. The use of specific electrode-catalyst and precisely defined value of working potentials allow matrix interference to be avoided. Therefore, the electrochemical sensor platforms offer a remarkable sensitivity for trace levels determination.
Repeatability and accuracy	Electrochemical sensors possess an excellent repeatability and accuracy. Once calibrated to a known concentration, the sensor device will provide an accurate measurement of a target analyte, that is repeatable.
Low sample volume and minimum pre-treatment	Low sample volume requirement with no prior treatment of the samples is another big advantage. The principle of operation of the electrochemical sensors allows procedures of the sample preparation to be kept to a minimum. The electrochemical method only requires appropriate sample dilution followed by immediate analysis.
Fast response	Important aspect is that electroanalysis is much simpler and requires less time in comparison with the more commonly used analytical methods. The electrochemical sensors provide a rapid response within a few seconds.
Inherent miniaturization	Electrochemical sensor is an ideal, attractive candidate for miniaturized analytical system, as it has high compatibility with modern micro- and nanofabrication technologies. Due to the ability to easily integrate electrodes in microchips, electrochemical sensor is actually easy to be miniaturized. Moreover, the analytical sensitivity is not compromised by miniaturization.
Low cost and power requirements	Ultra-low power consumption, easy-to-carry instruments and cost-effectiveness are among the most important features of electrochemical sensors.
Real time and on-site detection	The electrochemical sensor is a system that simultaneously meets all the key requirements for an on-site sensing.
Wireless network	Most of the advanced electrochemical sensors are equipped with the technology allowing them to be used as a part of a wireless network, i.e. they can be connected to laptops, tablets or smartphones.
**Disadvantages**	
Non-specific adsorption and biofouling	The non-specific adsorption of proteins on the electrode surface is persistent problem that negatively affects electrochemical sensors. This phenomenon occurs because of physisorption and can decrease the sensor’s performance – it results in higher background signal and affects the reliability of sensors. On the other hand, electrochemical reaction products tend to accumulate at the electrode surface leading to loss of catalytic activity and hence the sensor response decreases with time.
pH-dependence of electrode signal	Since the proton (H^+^) participates directly in the electrochemical process, pH is a key factor affecting the signal intensity. Therefore the sample analysis should be performed in a buffer solution at an optimal pH-value.
Device-to-device reproducibility	The sensing performance is highly dependent on the electrode surface morphology and may differ from device-to-device even though the catalytic-active electrode material originates from the same fabrication protocol.
